# Early Enzyme-Alginogel Treatment Improves Healing and Reduces Complications, Scarring, and Healthcare Burden in Children with Mild-to-Moderate Burns: A Retrospective Real-World Study

**DOI:** 10.3390/children13080994

**Published:** 2026-07-27

**Authors:** Biagio Nicolosi, Emanuele Buccione, Hamilton Dollaku, Benedetta Virginia Difalco, Eleonora Salutini, Alessandra Martin, Martina Certini, Sara Cappelli, Alessia Spano, Gianluca Castiello, Flavio Facchini, Guido Ciprandi

**Affiliations:** 1 Health Professions Department, Meyer Children’s Hospital IRCCS, 50139 Florence, Italy; benedetta.difalco@meyer.it (B.V.D.); eleonora.salutini@meyer.it (E.S.); 2 AISLeC, Italian Nursing Association of Wound Care, 47923 Rimini, Italy; gianluca.castiello@grupposandonato.it; 3 Pediatric Intensive Care Unit, Local Health Authority 3 of Pescara, 65124 Pescara, Italy; emanuele.buccione@asl.pe.it; 4 Cardiac Rehabilitation Unit, IRCCS Don Gnocchi Hospital, 50143 Florence, Italy; hdollaku@dongnocchi.it; 5 Simple Departmental Organizational Structure of Plastic Surgery and Burn Center, Meyer Children’s Hospital IRCCS, 50139 Florence, Italy; alessandra.martin@meyer.it (A.M.); martina.certini@meyer.it (M.C.); flavio.facchini@meyer.it (F.F.); 6 Bachelor’s Degree Program in Nursing, School of Human Health Sciences, University of Florence, 50139 Florence, Italy; sara1.cappelli@uslcentro.toscana.it (S.C.); alessia.spano2@edu.unifi.it (A.S.); 7 Cardiovascular Area and Adult & Pediatric Intensive Care, IRCCS Policlinico San Donato, 20097 San Donato Milanese, Italy; 8 Complex Unit of Plastic Surgery, University of Padua, 35128 Padua, Italy; guido.ciprandi@unipd.com

**Keywords:** pediatric burns, mild-to-moderate burns, enzyme alginogel, sodium hypoclorite, wound healing, burn wound management, early treatment, scar outcomes

## Abstract

**Highlights:**

**What are the main findings?**

- Early treatment with enzyme alginogel was associated with faster healing, fewer local complications, and better early scar outcomes than the historical NaOCl-based initial treatment strategy in children with mild-to-moderate burns.
- Children receiving enzyme alginogel required fewer follow-up visits and none underwent early surgery, despite a higher proportion of deep burns in this group.

**What are the implications of the main findings?**

- Early topical management may influence burn wound progression, healing, and early scar development in pediatric patients.
- Prospective randomized studies are needed to determine the role of enzyme alginogel within standardized treatment pathways for pediatric burns.

**Abstract:**

Background/Objectives: Pediatric mild-to-moderate burns require careful management in the early phase, yet evidence guiding optimal initial dressing selection remains limited. Although topical medications and specialized dressings are routinely used, a universally accepted standard of care is lacking. Methods: We conducted a 1-year, real-world, retrospective, comparative, single-center study evaluating whether treatment with enzyme alginogel (Flaminal^®^) or NaOCl 0.05% antiseptic in the first 24 h led to different outcomes in terms of healing time, lesion progression, complication occurrence, scar quality, and burden of care. Medical records of 80 children with mild-to-moderate burns (Total Body Surface Area < 15%) treated between January and December 2024 were analyzed. Results: Despite a higher proportion of deep burns, early treatment with enzyme alginogel was associated with a 6.17-day [95% Confidence Interval: 4.71–7.63 days] shorter healing time (*p* < 0.001), with no observed lesion worsening or need for early surgery. The complication rate was 14% in the enzyme alginogel group, while all patients receiving antiseptic dressing had at least one complication (*p* < 0.001). Patients treated with enzyme alginogel also showed better Vancouver Scar Score [2.95 points, 95% Confidence Interval: 2.33–3.23, *p* < 0.001] and fewer follow-up visits, supporting a direct relationship between healing time and scar outcomes, and suggesting a potential reduction in the burden of care. Conclusions: Early management with alginogel favored healing, reduced complications, improved scar outcomes, and reduced burden of care in comparison to the initial treatment with NaOCl-based antiseptic dressings followed by treatment change when clinically indicated. Prospective, comparative, randomized studies are needed to confirm these findings.

## 1. Introduction

Pediatric burns represent one of the leading sources of non-fatal injury, substantially contributing to disability-adjusted life years [1,2,3]. Mild-to-moderate burns generally correspond to first-degree superficial, superficial partial-thickness (IIA), or deep partial-thickness (IIB) burns involving a limited total body surface area (TBSA), and are usually managed conservatively [4]. Routine practice envisages application of topical medications and specialized dressings that can create an optimal wound environment to favor timely re-epithelialization—ideally within 18–21 days—and reduce the probability of complications such as infection, hypertrophic scarring or contracture [5,6,7,8].

The ideal dressing should be easy to apply and remove, comfortable for the patient, and be cost-effective, while simultaneously promoting uncomplicated healing [7,9,10]. Silver-based products, antimicrobial ointments, biosynthetic membranes, and topical antiseptic solutions represent viable approaches for the management of mild-to-moderate pediatric burns, but no consensus exists yet on a universally accepted optimal dressing that could represent the standard of care [10,11,12,13,14]. Rather, medication selection is still highly dependent on costs, availability, required frequency of dressing change, and institutional familiarity. This reflects the persistent uncertainty surrounding the optimal management of mild-to-moderate pediatric burns. Such uncertainty is clinically impactful specifically in the early phases of wound management, when treatment decisions can determine wound progression, re-epithelialization time, and long-term scar outcomes [7,12,15].

Although several advanced dressings are currently available for the management of partial-thickness burns, recent systematic reviews have concluded that the available evidence remains insufficient to support the routine use of one dressing over another in all clinical settings. Treatment selection should therefore be guided by burn characteristics, patient-related factors, and clinical experience. Recent evidence has also suggested potential benefits of alginate-based dressings in promoting wound healing; however, further clinical studies are needed to better define their role in everyday pediatric burn care [7,9].

Topical application of sodium hypochlorite (NaOCl)-based solutions (e.g., Dakin’s solution) is a low-cost approach providing wide-spectrum bactericidal activity [10,16,17,18]. This represents a pragmatic first-line option in the early treatment of mild-to-moderate burns in many healthcare centers and is applied to achieve rapid microbial control and reduce treatment complexity. Subsequently, clinical reassessment at short-term follow-up is planned to define timely modification or escalation to alternative advanced dressings in case of wound progression or suboptimal healing [18,19].

More recently, enzyme alginogels have been introduced for the management of chronic and acute wounds [20,21,22,23]. Enzyme alginogels are hydroactive alginate-based matrices incorporating an enzymatic antimicrobial system designed to maintain a moist wound environment, support autolytic debridement, absorb excess exudate, and provide broad-spectrum antimicrobial activity with relatively low cytotoxicity, thereby fulfilling several characteristics of an ideal dressing [6]. In partial-thickness burns, direct comparative studies have demonstrated favorable clinical outcomes of enzyme alginogel compared with 1% silver sulfadiazine, including comparable healing outcomes and a reduced need for dressing changes. Subsequent studies have reported similar long-term outcomes; however, interpretation of direct comparisons with silver sulfadiazine requires caution because additional topical antimicrobial treatments were incorporated into the comparator treatment pathway during follow-up [6]. These findings suggest that enzyme alginogels may be advantageous in the management of mild-to-moderate burns in children, where minimizing painful dressing changes and preserving function are particularly important objectives [6,7].

However, evidence on the use of enzyme alginogels in pediatric burns is still limited, particularly in the real-world setting and in the management of the critical first 24 h after injury, when burn wound progression and inflammatory dynamics influence healing and scar outcomes. Moreover, comparative clinical data evaluating early alginogel use against other commonly adopted topical treatments are still lacking in pediatric populations.

The aim of the present study was to evaluate whether early treatment with an enzyme alginogel could represent an effective alternative to the historical local standard of care (SoC) at our center, consisting of initial treatment with NaOCl-based dressings, followed by treatment modification according to lesion evolution, with subsequent treatment modification according to lesion evolution and clinical reassessment. The overarching purpose of the study is to contribute to the definition of standard clinical practices for early treatment of children with burn injuries and the development of more effective, individualized care pathways.

## 2. Materials and Methods

### 2.1. Study Design

This was a single-center retrospective observational real-world study comparing two non-randomized groups of patients classified according to the initial topical treatment after presentation to the Emergency Department of Meyer Children’s University Hospital IRCCS, Florence, Italy. The study started on 1 July 2025, and was completed on 31 October 2025.

### 2.2. Ethical Considerations

The study was conducted in accordance with Good Clinical Practice principles and applicable data protection regulations (Italian Privacy Code, D.lgs. 196/2003 as amended by D.lgs. 101/2018; EU Regulation 2016/679—GDPR). The study was approved by the Pediatric Ethics Committee of the Tuscany Region with protocol number 126/2025 on 10 June 2025. Given the retrospective observational design, a waiver of informed consent was applied pursuant to Article 110 of the Italian Privacy Code. The study complied with the Declaration of Helsinki and ISO 14155:2020 [24].

The study is reported in accordance with the “Strengthening the Reporting of Observational Studies in Epidemiology (STROBE)” guidelines for observational studies [25].

### 2.3. Patient Selection

Clinical data were retrospectively extracted from the institutional electronic medical record system. The study considered 80 consecutive pediatric patients (0–18 years) presenting to the Emergency Department of the center between 1 January 2024 and 31 December 2024 with a documented burn injury and/or undergoing scar management follow-up. Eligible patients were also required to have a documented burn injury with TBSA < 15% (this precautionary threshold was considered representative of a mild-to-moderate burn) and complete clinical records.

To better contextualize burn severity classification in pediatric patients, it is important to acknowledge that TBSA-based severity thresholds are age-dependent, reflecting differences in body surface distribution and physiological response to burn injury. In pediatric populations, the definition of mild, moderate, and severe burns is not universally standardized and varies across guidelines, particularly in younger children [7,14]. For transparency and clinical contextualization of the inclusion criteria adopted in this study (TBSA < 15%), a summary of commonly used TBSA-based severity classifications stratified by age is provided in Table 1.

Because severity thresholds vary according to age, no single TBSA value can universally define mild-to-moderate burns throughout childhood. Consequently, a pragmatic threshold of <15% TBSA was adopted to include patients who, across the pediatric age spectrum, are predominantly managed with conservative local treatment rather than immediate major burn protocols. This approach was intended to increase the clinical homogeneity of the study population while maintaining external validity for routine pediatric burn care.

Patients were excluded if essential data regarding burn severity, treatments received, or scar-related outcomes were incomplete or missing. All included patients had complete clinical records meeting eligibility criteria.

### 2.4. Treatment Groups and Procedure

Included patients were classified into two treatment group presentations. Group A included patients treated with NaOCl-based antiseptic dressings 0.05%, which represented the prevalent initial local SoC at our center during the study period. Group B included patients treated with topical enzyme alginogel (Flaminal^®^, Flen Health NV, Kontich, Belgium), which consists of hydrated alginate polymers embedded in a polyethylene glycol (PEG)/water matrix containing a patented antimicrobial enzymatic complex [26]. In routine practice, NaOCl-based dressings were not necessarily intended as definitive treatment throughout the entire healing process. Rather, they represented the most frequently adopted first-line antiseptic approach in the acute phase, followed by wound reassessment and possible transition to other advanced dressings according to lesion depth, exudate, wound progression, and clinician judgement.

Because of the retrospective observational design, treatment allocation was not randomized. Patients were classified according to the initial topical treatment received within the first 24 h after presentation, reflecting routine clinical practice at our center. The choice of the initial topical treatment was made by the attending clinician in accordance with the local treatment strategy in use at the time of presentation.

At ED presentation, devitalized tissue and non-viable blistered skin were removed when present. After wound cleansing, topical treatment was initiated according to the therapeutic strategy adopted by the attending clinician. Wounds in the NaOCl group were covered with cotton gauze pads secured with a light fixation bandage, whereas alginogel-treated wounds were covered with paraffin-impregnated gauze followed by a light securing bandage. Dressings were routinely renewed and wounds reassessed within the first 72 h—generally at 24 h in the NaOCl group and at 48 h in the alginogel group—to monitor early clinical evolution and detect possible burn wound progression. Regardless of the scheduled dressing change, all patients underwent routine clinical monitoring during the early post-burn period. Additional clinical assessments were performed whenever clinically indicated to identify burn wound progression, increasing edema, vascular compromise, or other conditions requiring earlier dressing removal, treatment modification, or surgical evaluation. According to the institutional burn care protocol, the need for surgical management was assessed during the initial evaluation and early follow-up (within 72 h after injury). The indication for surgery was established by the multidisciplinary burn team according to routine clinical practice, taking into account burn depth, the evolution of the wound during the first days after injury, tissue viability, anatomical site, TBSA involvement, and the expected potential for spontaneous healing. Patients who did not meet the clinical criteria for surgery during this period continued conservative treatment until complete healing. When surgery was indicated, early excision of non-viable tissue was performed, followed by definitive wound coverage using the reconstructive technique considered most appropriate for the individual lesion, including split-thickness skin grafting, dermal substitutes, or a combination of these approaches.

Following early reassessment, treatment was either continued or switched to an alternative dressing according to the clinical findings.

For NaOCl-treated wounds, alternative or subsequent treatments included gelling fiber with silver, sodium hyaluronate cream, collagenase combined with sodium hyaluronate, or enzyme alginogel, according to wound characteristics and clinical evolution. Therefore, the comparison between groups should be interpreted as a comparison between initial treatment strategies applied within the first 24 h, rather than as a comparison between fixed treatment protocols maintained unchanged until complete healing. Treatment decisions following the initial conservative burn management (e.g., continuation of topical therapy or indication for surgical intervention) were based on burn depth, clinical evolution, and standard institutional practice.

### 2.5. Data Collection and Clinical Assessment

Collected variables included age, sex at birth, burn etiology, location and severity, lesion evolution, healing time (i.e., days from ED presentation to complete wound re-epithelialization, as clinically documented in the medical records), scar outcomes assessed using the Vancouver Scar Scale (VSS) [27] one month after complete wound re-epithelialization and local complications (NERDS, inflammation, exudate, maceration, lesion borders) [28]. Local complications were assessed based on predefined clinical criteria derived from routine clinical documentation. NERDS criteria were identified according to the presence of non-healing, increased exudate, red friable tissue, debris, and smell. Inflammation, exudate, and maceration were categorized based on clinical judgement documented in the medical records, following standard institutional wound assessment practices. Given the retrospective design, no centralized adjudication of outcomes was performed.

Burn severity was assessed according to total body surface area involvement (TBSA, %) and burn depth, as documented in the medical records. TBSA was estimated at Emergency Department presentation using the Lund and Browder chart. Burn depth was clinically assessed on the basis of wound appearance, color, moisture, capillary refill, sensitivity, and clinical evolution, as recorded by the attending clinician. Burns were classified as first-degree superficial burns, superficial partial-thickness burns (IIA), deep partial-thickness burns (IIB), or full-thickness burns (III). Given the retrospective design, burn-depth assessment was based on routine clinical documentation, and no centralized or blinded adjudication was performed. For each patient, the overall TBSA and the predominant burn depth documented in the medical records were collected. The extent (%TBSA) and anatomical distribution of individual burn-depth components were not routinely recorded.

### 2.6. Objectives and Endpoints

The primary objective was to compare the effectiveness of NaOCl (Group A) and enzyme alginogel (Group B) initial treatments in the management of mild-to-moderate pediatric burns. Primary endpoints included clinical healing time and early lesion evolution within the first 72 h or at the first clinical reassessment.

Secondary objectives included the evaluation of local complications and dressing management (need for early dressing change and subsequent treatments), scar evaluation according to VSS total score and sub-scores, predictors of lesion worsening within 72 h, and healthcare burden assessed through the number of follow-up visits. Exploratory analyses were also conducted to evaluate the influence of clinical and demographic predictors on the outcomes of intertest (need for early surgery, multiple drivers of healing time, of VSS total score and sub-scores, and early medication change).

### 2.7. Statistical Analysis

Statistical analyses were performed comparing Group A and Group B. Continuous variables were reported as mean and standard deviation (SD) and median with interquartile range (IQR), while categorical variables were expressed as absolute and relative frequencies. Age and TBSA were analyzed both as continuous and categorical variables. Normality of continuous data was assessed using the Shapiro–Wilk test.

Baseline differences between groups were evaluated using the Wilcoxon test for continuous variables and Fisher’s exact test for categorical variables. The primary endpoint of clinical healing time was compared between groups using the Welch’s *t*-test, while early lesion evolution within 72 h was analyzed using Fisher’s exact test. Secondary outcomes, including local complications, dressing management, and VSS scores, were analyzed using descriptive statistics together with Wilcoxon or Fisher’s exact tests as appropriate. Predictors of early lesion worsening within 72 h were evaluated using a multivariate logistic regression model. Exploratory analyses were conducted using multivariate regressions, linear regressions for healing time and VSS score variability, logistic regressions for complications and early dressing change, and negative binomial regression to analyze healthcare burden measured by the number of follow-up visits.

Multivariate regression models accounted for potential confounding factors, including age, TBSA, burn depth, and burn etiology. Burn depth was reported descriptively using the four recorded clinical categories. In multivariable analyses, superficial partial-thickness (IIA) and deep partial-thickness (IIB) burns were combined into a single second-degree category because of the small number of observations in the individual subcategories, particularly in Group B, thereby reducing the risk of unstable and imprecise model estimates.

A forward selection approach guided by clinical criteria was used for model building, with predictors sequentially included and retained according to clinical relevance and improvement in model fit, assessed using likelihood ratio tests and changes in the Akaike Information Criterion (AIC).

All statistical tests were two-sided with alpha equal to 0.05, and 95% confidence intervals (CI) were reported. No data was missing among included patients; thus, no technique was applied for the management of missing values.

No formal a priori sample size calculation was performed because the study included all consecutive eligible patients identified during the predefined study period.

(version 4.3.3; R Foundation for Statistical Computing, Vienna, Austria) and RStudio (version 2023.12.1+402; Posit Software, PBC, Boston, MA, USA).

## 3. Results

### 3.1. Study Population

**Figure 1:**
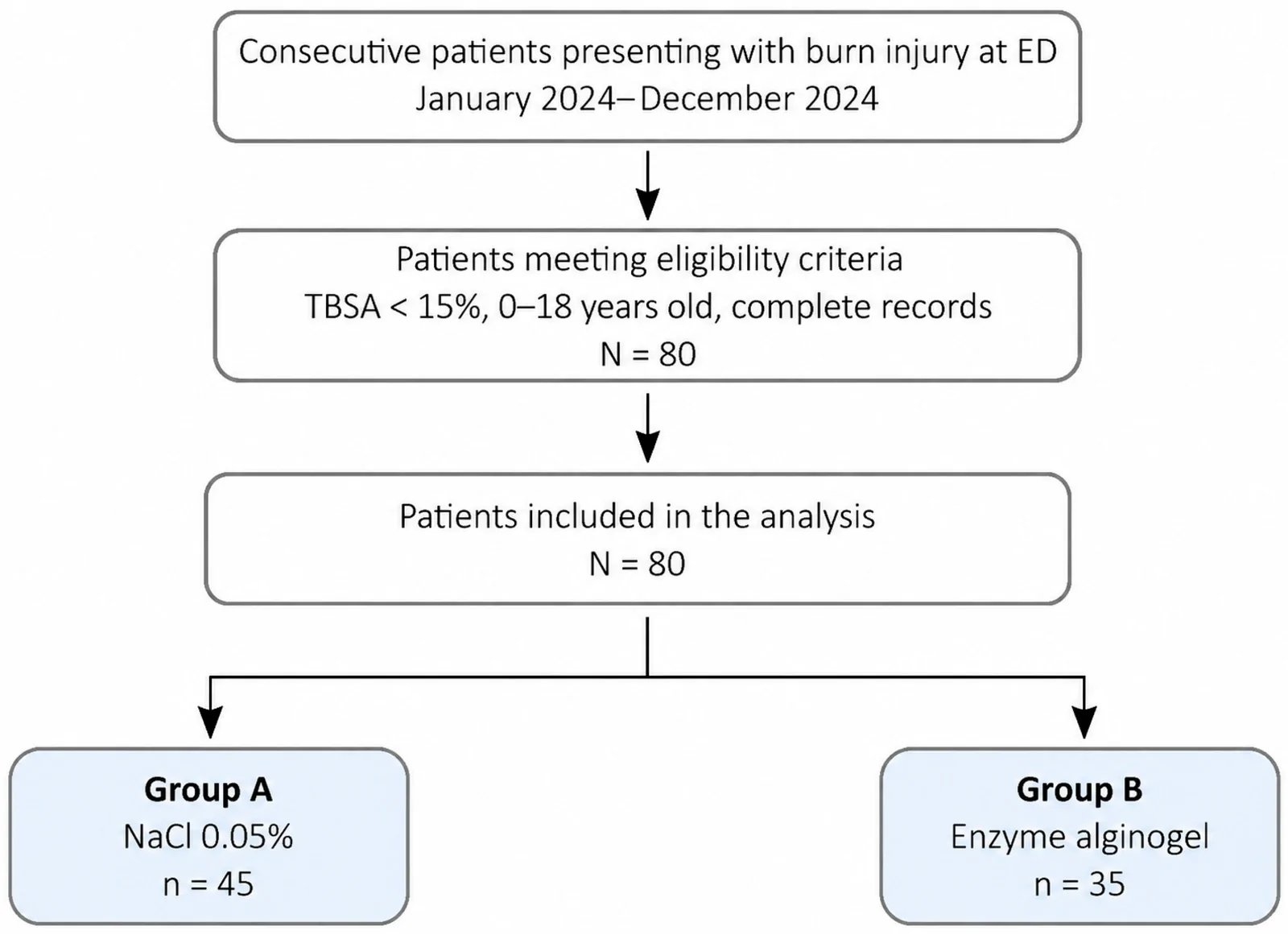
Flow diagram of patient selection and treatment allocation. Consecutive pediatric patients presenting with burn injury at the Emergency Department (ED) between January 2024–December 2024 were retrospectively screened for eligibility. Patients meeting inclusion criteria (age 0–18 years, TBSA < 15%, and complete clinical records) were included in the analysis (n = 80). All included patients had complete clinical data; therefore, no exclusions due to missing data were required. Included patients were classified according to the topical treatment applied within the first 24 h into Group A (NaOCl 0.05%, n = 45) and Group B (enzyme alginogel, n = 35).

Eighty patients (53.8% females) were included in the analysis, 45 belonging to Group A and 35 to Group B. Figure 1 shows the patient selection process and treatment allocation.

Patient demographic and clinical characteristics are detailed in Table 2.

Median age was 8.0 years (IQR 3.0–12.0 y; range 5–16 y). Burn injuries were predominantly superficial (51.3%) and were caused by liquids (72.5%), scalds (15.0%) or flames (12.5%), and mostly occurred in multiple regions (41%) or upper limbs (27%). Burn depth distribution differed significantly between treatment groups (Fisher–Freeman–Halton exact test, *p* = 0.033). Overall, first-degree superficial burns represented the most frequent injuries (41/80, 51.3%), followed by full-thickness (III) burns (27/80, 33.8%), superficial partial-thickness (IIA) burns (8/80, 10.0%), and deep partial-thickness (IIB) burns (4/80, 5.0%). Full-thickness burns were proportionally more frequent in Group B (17/35, 48.6%) than in Group A (10/45, 22.2%), whereas first-degree superficial burns predominated in Group A (25/45, 55.6%) compared with Group B (16/35, 45.7%). The higher proportion of full-thickness burns in Group B is consistent with the baseline distribution of patients managed according to routine clinical practice in this retrospective observational study rather than treatment allocation based on a predefined study protocol. Overall, 59 of 80 patients (73.8%) achieved complete healing with conservative treatment alone, whereas 21 patients (26.3%) underwent early surgical management within 72 h according to the institutional burn care protocol. Consistent with this protocol, no patient who did not require surgery during the initial 72-h assessment subsequently underwent surgical treatment during follow-up.

### 3.2. Primary Objectives

**Figure 2:**
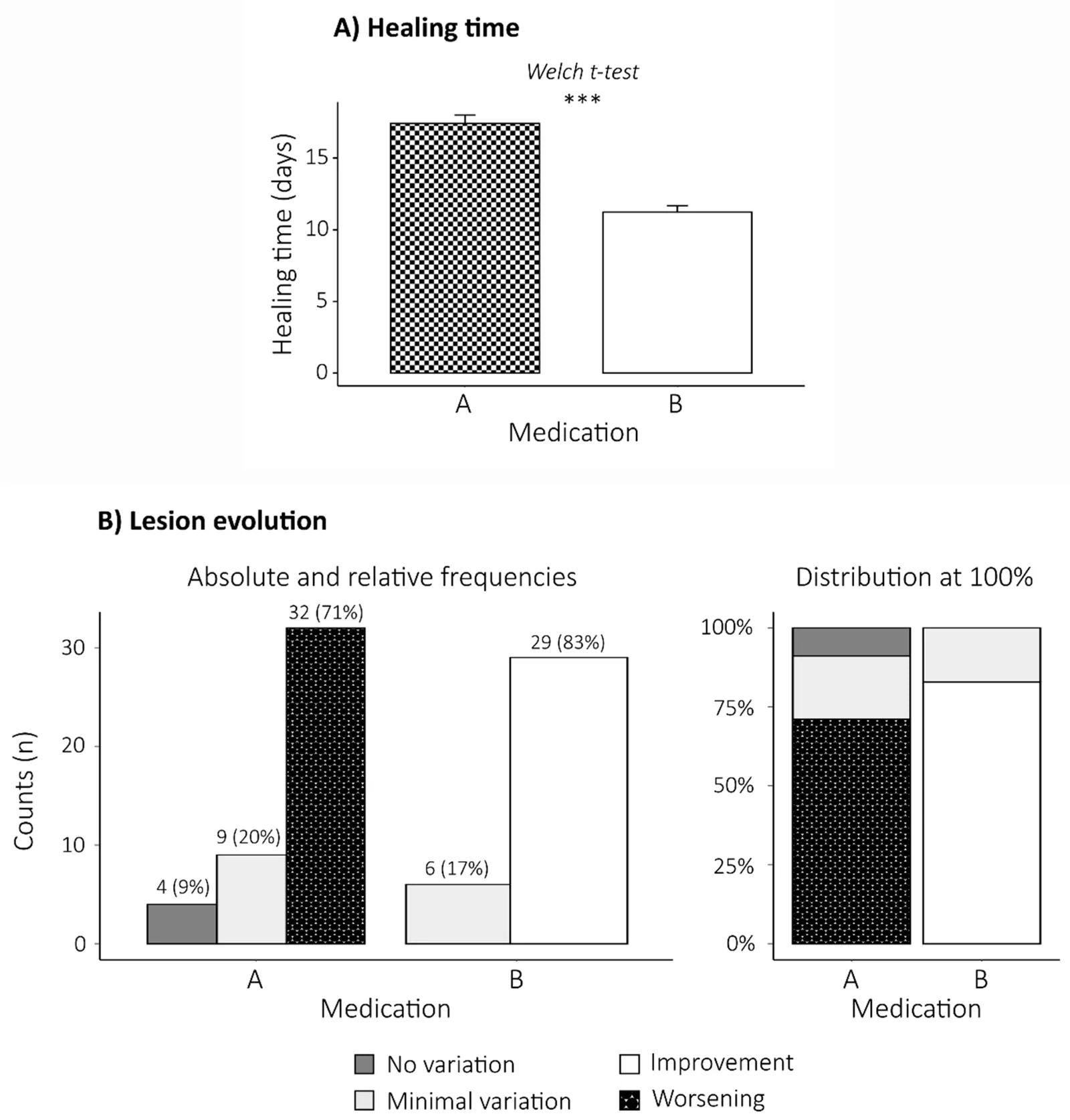
Primary outcomes. (**A**) Mean ± SD in healing time (days) between Group A (NaOCl solution 0.05%) and Group B (alginogel). (**B**) Lesion evolution (no variations, minimal variation, improvement, and worsening) within the first 72 h or first assessment in the two groups; left panel reports absolute frequency (counts, n) within groups with percentage shown above bars; right panel reports distributions normalized to 100%. Asterisks indicate a *p*-value < 0.001.

In Group A, healing time ranged from 9 to 27 days (mean ± SD: 17.4 ± 4.0 days), and in Group B from 6 to 16 days (mean ± SD: 11.2 ± 2.6 days) (Table 3), resulting in a significant (*p* < 0.001) mean difference of 6.17 days between treatments [95% CI: 4.71–7.63 days] (Figure 2A).

When healing time was examined according to burn depth, enzyme alginogel was consistently associated with shorter healing times among patients with first-degree superficial burns (9.7 vs. 16.4 days) and full-thickness (III) burns (12.4 vs. 20.5 days). Superficial partial-thickness (IIA) burns showed similar healing times between treatment groups (13.5 vs. 14.5 days). Deep partial-thickness (IIB) burns were observed only in Group A, precluding any direct comparison for this subgroup. Therefore, analyses for IIA and IIB burns should be considered descriptive because of the limited sample size (Table S1; Figure S1).

Lesion evolution significantly differed between treatments (Fisher’s exact test, *p* < 0.001), as most lesions worsened in Group A (71.11%), with the other cases showing no change (8.89%) or minimal change (20.0%). In contrast, lesions in Group B mostly improved (82.86%), with the remaining cases showing minimal change (17.14%) and no worsening (Figure 2B).

### 3.3. Secondary Objectives

Local complications and dressing management strategies are summarized in Table 4.

NERDS were observed in all patients in Group A and in five patients in Group B (14.29%), resulting in a significantly different distribution (Fisher’s exact test, *p* < 0.001). This finding should be interpreted cautiously, as it may partly reflect differences in clinical documentation practices and the retrospective identification of NERDS criteria, rather than a true uniform clinical occurrence. Accordingly, the evolution of inflammation, exudate, maceration, and lesion borders significantly differed between Group A and Group B, favoring treatment with enzyme alginogel (*p* < 0.01). None of the patients treated with enzyme alginogel underwent medication change after the first reassessment. Conversely, 44 of 45 patients initially treated with NaOCl required a subsequent medication change, indicating that NaOCl mainly functioned as the prevalent initial local SoC rather than as a definitive treatment pathway. Subsequent treatments most frequently included gelling fiber with silver (60.0%), collagenase combined with sodium hyaluronate (22.2%), sodium hyaluronate cream (8.9%), and enzyme alginogel (6.7%), with some differences according to burn depth (Table S2).

Total VSS score ranged from 1 to 7 (mean ± SD: 4.4 ± 1.2) in Group A and from 0 to 3 (mean ± SD: 1.4 ± 0.9) in Group B (Table 5), resulting in a significant difference (Wilcoxon test, *p* < 0.001); mean difference in VSS score between treatments was 2.95 [95% CI: 2.33–3.23]. Outcomes referring to VSS sub-scores indicated significant differences (*p* < 0.001) between groups favoring Group B (Table 5).

After combining superficial partial-thickness (IIA) and deep partial-thickness (IIB) burns into a single second-degree category, second-degree burn depth was the only variable significantly associated with lesion worsening within 72 h (OR 4.24, 95% CI: 1.05–17.14; *p* = 0.043). Full-thickness burn depth and TBSA were not significantly associated with lesion worsening. Given the limited explanatory performance of the model and the wide confidence interval, these findings should be considered exploratory.

**Figure 3:**
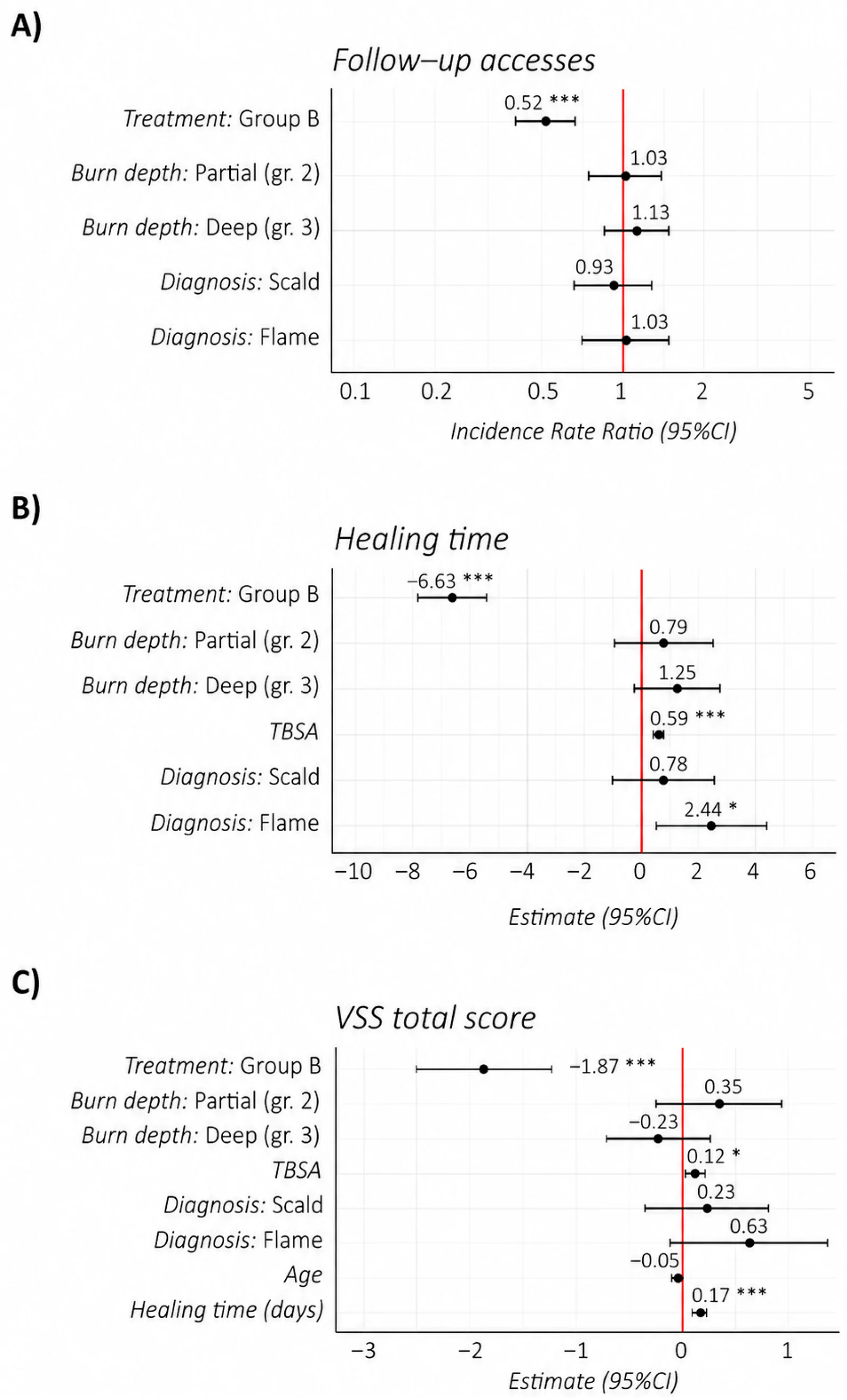
Results of the multivariable regression analyses. (**A**) Negative binomial regression model estimating the number of follow-up visits (reported as incidence rate ratios, IRRs); (**B**) linear regression model estimating healing time (days); (**C**) linear regression model estimating total Vancouver Scar Scale (VSS) score. Black dots represent the estimated regression coefficients (IRRs in panel (**A**) and regression estimates in panels (**B**,**C**)), and horizontal bars indicate the corresponding 95% confidence intervals (95% CI). Red vertical lines indicate the null effect (IRR = 1 in panel (**A**); estimate = 0 in panels (**B**,**C**)). Asterisks denote statistical significance (* *p* < 0.05; *** *p* < 0.001).

Healthcare burden, assessed according to the number of follow-up visits, showed a favorable profile of Group B (median [IQR]: 3.0 [2.0–3.0]) in comparison to Group A (median [IQR]: 5.0 [4.0–5.0]). This result was confirmed by the regression model showing that treatment was the only significant predictor of the number of follow-up visits (*p* < 0.001, R^2^_Nagelkerke_: 0.75) (Figure 3A).

### 3.4. Exploratory Outcomes

No patient in Group B underwent early surgery within 72 h, while 47% of patients in Group A did (Fisher’s exact test, *p* < 0.001), especially those with Grade 3 burns (Table S2). This difference should be interpreted with caution, as it may reflect underlying clinical decision-making processes and the potential cumulative effect of uncontrolled baseline differences, rather than the isolated effect of the initial topical treatment. In particular, this finding may be influenced by confounding by indication, whereby patients perceived at higher risk of progression may have been managed more aggressively from the early phase. Surgical interventions were predominantly observed in patients with deeper burns, consistent with standard clinical practice (Table S2). The multivariate regression model for healing time resulted in the explanation of a substantial proportion of the observed variability (R^2^ = 0.71)—healing time significantly decreased with enzyme alginogel treatment (*p* < 0.001) while being directly associated with TBSA (*p* < 0.001) and flame-derived burns (*p* = 0.02) (Figure 3B). The final multivariate model for VSS total score explained 82% of the variability (R^2^ = 0.82): lower scores significantly associated with alginogel-based treatment (*p* < 0.001), while a direct association was found with healing time (*p* < 0.001) and TBSA (*p* = 0.02) (Figure 3C). Available predictors were not sufficient to define regression models explaining a substantial variability of VSS sub-scores or the early medication change.

## 4. Discussion

Our findings highlight the critical role of early lesion management in shaping healing trajectories of mild-to-moderate pediatric burns. The pragmatic adoption of a <15% TBSA inclusion criterion should be interpreted in light of the absence of universally accepted pediatric burn severity thresholds. Although severity classifications vary according to age, this cut-off allowed the present study to focus on the population in whom early topical management is most clinically relevant, while limiting the heterogeneity associated with more extensive burns requiring substantially different treatment pathways.

While early application of NaOCl-based antiseptic dressings is still common in routine medical practice due to easy access, antimicrobial activity, and affordable costs [10,29], our findings suggest that this approach mainly reflected a prevalent initial local SoC rather than a complete treatment pathway. Indeed, most patients initially treated with NaOCl required subsequent transition to advanced dressings after early reassessment. Conversely, early enzyme alginogel treatment was associated with faster healing, more favorable early lesion evolution, fewer local complications, better scar outcomes, and a reduced burden of care.

The mean healing time observed in the enzyme alginogel group was 11.2 days, corresponding to a 6.17-day reduction compared with NaOCl-based treatment. Importantly, the association between enzyme alginogel and shorter healing time remained evident after accounting for burn depth, which is a well-established determinant of healing. Nevertheless, because burn-depth distribution differed between groups, residual confounding cannot be excluded despite multivariable adjustment and stratified analyses. This finding is particularly relevant because the alginogel group included a higher proportion of deep burns. The shorter healing time is clinically relevant, as re-epithelialization within the observed time frame of 6–16 days did not exceed the 18–21-day window usually considered for uneventful lesion resolution, thus possibly helping to reduce the risk of complications and unfavorable scar evolution [7,8]. Accordingly, only 14% of patients treated with enzyme alginogel met NERDS criteria compared with the 100% rate retrospectively identified in the NaOCl group, which can be interpreted in light of the pathophysiology of burn wounds. Burns are characterized by skin barrier disruption, devitalized tissue, sustained inflammation, increased exudate, local hypoxia, and bacterial proliferation, all of which can promote critical colonization and unfavorable lesion progression [30,31]. In this context, NaOCl-based antiseptic treatment may provide antimicrobial activity but may be less effective in addressing key principles of Wound Bed Preparation, particularly debridement, exudate control, and modulation of the local inflammatory environment [32].

Experimental studies have also reported concentration- and exposure-dependent cytotoxic effects of sodium hypochlorite on fibroblasts and keratinocytes, which may contribute to delayed wound healing. This should be considered when interpreting our findings. However, in our center NaOCl represented the historical initial standard of care rather than a definitive treatment, and patients routinely underwent early reassessment with treatment modification when clinically indicated. Therefore, our results should be interpreted as reflecting different initial management strategies rather than the effects of prolonged NaOCl exposure. Nevertheless, a possible contribution of NaOCl-related cytotoxicity cannot be excluded and remains a potential source of residual confounding [17,33].

By contrast, enzyme alginogel may support a more balanced wound microenvironment through autolytic debridement, antimicrobial protection, absorption of excess exudate, and maintenance of a moist environment favorable to epithelial migration [26,34].

Our results also support the view that early wound management can significantly influence lesion evolution within the first 72 h [7,12,35]. Patients treated with NaOCl showed a high rate of lesion worsening and frequently required early surgery, whereas no worsening and no early surgical intervention were observed in the enzyme alginogel group. As expected, deeper burns were more likely to require surgical management. However, the absence of early surgery in the alginogel group, despite a higher proportion of deep lesions, suggests a potential role of early topical management in limiting wound progression. This finding should be yet regarded as purely exploratory, as surgical decisions may have been influenced by wound severity, clinician judgement, and local management practices. The favorable early evolution observed with enzyme alginogel was also reflected in scar characteristics assessed one month after complete wound healing. VSS total score and sub-scores were lower in the alginogel group, and the multivariate model confirmed a direct association between healing time and VSS total score. This finding supports an association between faster re-epithelialization and more favorable early scar characteristics [36,37]. In addition, patients treated with enzyme alginogel required fewer follow-up visits, suggesting a reduced healthcare burden. This may have practical implications in pediatric burn care, where fewer dressing changes and visits may reduce pain, distress, family burden, and healthcare costs [38,39,40,41].

From a broader perspective, recent evidence indicates that, in the absence of a universally accepted standard of care, early treatment decisions for pediatric burns are often influenced by local protocols, resource availability, and clinician experience [14,19]. Real-world data from Italian burn centers similarly confirm substantial variability in antiseptic use and dressing selection, particularly during the early phase of management [14]. The present findings should therefore be interpreted within this context of clinical variability and support the need for more standardized, depth-driven treatment frameworks.

Compared with previous studies evaluating Flaminal^®^ in adult patients with burns of comparable severity, the mean healing time observed in our cohort was shorter [6,11]. This difference can be partly attributed to the inclusion of pediatric patients into the present study, who typically show more efficient physiological processes for re-epithelialization and tissue remodeling [40], but this interpretation would require direct comparison. The observed beneficial effects are consistent with the functional properties of Flaminal^®^, which combines an enzyme-based antimicrobial system with an alginate matrix able to absorb excess exudate while preserving a moist wound environment. This combination may help protect wound edges and epithelial cells and facilitate epithelial migration, whereas dryness and scab formation can create mechanical barriers that delay wound closure [7,26,34].

### Limitation

This study has several limitations that should be considered when interpreting the findings. First, no formal a priori sample size calculation was performed. The study included all consecutive patients meeting the eligibility criteria during the predefined study period; therefore, the sample size was determined by the number of eligible patients rather than by a prespecified statistical hypothesis. Although several comparisons reached statistical significance, the limited sample size may have reduced the precision of some estimates, particularly in subgroup analyses involving less frequent burn-depth categories.

Second, the retrospective observational design imposed inherent methodological constraints. Treatment allocation was not randomized but reflected routine clinical practice, and treatment decisions were based on clinical judgement within the institutional burn care pathway. Consequently, selection bias and confounding by indication cannot be excluded, despite adjustment for relevant clinical variables in the multivariable analyses. Likewise, burn depth was assessed according to routine clinical evaluation rather than by centralized or blinded adjudication and may therefore have been influenced by interobserver variability and by changes in wound appearance during the early post-burn period.

The retrospective nature of the study also limited the availability of some clinically relevant variables. The institutional database was developed primarily to support routine clinical care rather than research; therefore, although overall TBSA and the predominant burn depth were systematically recorded, TBSA according to individual burn-depth categories, the anatomical extent of each burn-depth component, and the presence of circumferential burns were not routinely documented and could not be analysed retrospectively.

In addition, the findings should be interpreted within the context of a specialized pediatric burn centre. Patient management, including the indication and timing of surgery, reflected the institutional multidisciplinary care pathway and the clinical resources available at our centre. Although this approach represents routine practice in our institution, treatment strategies and organizational pathways may differ across burn centres, potentially limiting the generalizability of the results.

Scar assessment was performed at a standardized follow-up visit one month after complete re-epithelialization. Although this ensured a uniform timing of evaluation, the follow-up period was not sufficient to assess definitive scar maturation. Therefore, the Vancouver Scar Scale results should be interpreted as early scar outcomes, since scar vascularity, pigmentation, pliability, and thickness continue to evolve over several months after wound healing.

Finally, patients were retrospectively classified according to the initial topical treatment received within the first 24 h after presentation. Subsequent treatment could be modified according to clinical evolution and routine reassessment. Consequently, the comparison should be interpreted as evaluating two initial treatment strategies within real-world clinical practice rather than two fixed therapeutic cohorts managed under standardized prospective conditions.

Prospective multicentre studies with larger patient populations and standardized treatment protocols are warranted to confirm these findings and to further define the role of enzyme-alginogel–based dressings in the management of mild-to-moderate pediatric burns.

## 5. Conclusions

In this retrospective real-world study, early use of enzyme alginogel was associated with improved clinical outcomes compared with the initial use of NaOCl-based antiseptic dressings, including shorter healing time, fewer complications, better scar outcomes, and a lower healthcare burden. These findings support the need for prospective randomized studies to confirm the observed associations.

Despite the inherent limitations of its retrospective design, this study suggests that early enzyme alginogel-based management may represent an effective option for mild-to-moderate pediatric burns. Notably, most patients initially managed with NaOCl required subsequent transition to other advanced dressings after early reassessment, whereas no treatment escalation was observed in the alginogel group. These findings support the potential role of early alginogel-based management in optimizing both short- and long-term outcomes in pediatric burn care. Future prospective, randomized comparative studies are warranted to confirm and extend these observations.

## Figures and Tables

**Table 1:** TBSA-based classification of burn severity in pediatric patients according to age groups.

Age Group	Mild Burn	Moderate Burn	Severe Burn
<2 years	<5% TBSA	5–10% TBSA	>10% TBSA
2–10 years	<10% TBSA	10–15% TBSA	>15% TBSA
>10 years	<10–15% TBSA	15–20% TBSA	>20% TBSA

**Table 2:** Demographic and clinical characteristics of included patients (n = 80). Group A refers to early dressing with NaOCl solution 0.05%; Group B refers to dressing with alginogel. Percentages are calculated by column.

Variable	Parameter	Treatment (n = 80)		Total	*p*-Value ^a^
		Group A n = 45 (56.25%)	Group B n = 35 (43.75%)		
Age (years)	Min/Max	0.6/16.0	0.5/16.0	0.5/16.0	0.95
	Med [IQR]	7.0 [5.0; 12.0]	8.0 [3.0; 12.0]	8.0 [3.0; 12.0]	
	Mean (std)	8.1 (4.7)	8.0 (5.3)	8.1 (4.9)	
	n (NA)	45 (0)	35 (0)	80 (0)	
Age class (years)	0–3	9 (20.0%)	12 (34.29%)	21 (26.25%)	0.08
	4–10	21 (46.67%)	8 (22.86%)	29 (36.25%)	
	11–18	15 (33.33%)	15 (42.86%)	30 (37.50%)	
Sex	Female	22 (48.89%)	21 (60.0%)	43 (53.75%)	0.32
	Male	23 (51.11%)	14 (40.0%)	37 (46.25%)	
Burn diagnosis	Liquid	35 (77.78%)	23 (65.71%)	58 (72.50%)	0.42
	Scald	6 (13.33%)	6 (17.14%)	12 (15.00%)	
	Flame	4 (8.89%)	6 (17.14%)	10 (12.50%)	
Burn depth	First-degree superficial	25 (55.56%)	16 (45.71%)	41 (51.25%)	0.03
	Superficial partial-thickness, IIA	6 (13.33%)	2 (5.71%)	8 (10.00%)	
	Deep partial-thickness, IIB	4 (8.89%)	0 (0.00%)	4 (5.00%)	
	Full-thickness, III	10 (22.22%)	17 (48.57%)	27 (33.75%)	
Burn site	Upper limb/hand	13 (28.89%)	9 (25.71%)	22 (27.5%)	0.91
	Lower limb	3 (6.67%)	3 (8.57%)	6 (7.50%)	
	Trunk	10 (22.22%)	8 (22.86%)	18 (22.5%)	
	Head/face	0 (0%)	1 (2.86%)	1 (1.25%)	
	Multiple regions	19 (42.22%)	14 (40.0%)	33 (41.00%)	
TBSA (%)	Min/Max	1.0/12.0	2.0/12.0	1.0/12.0	0.88
	Med [IQR]	8.0 [5.0; 10.0]	8.0 [4.5; 10.0]	8.0 [5.0; 10.0]	
	Mean (std)	7.2 (3.4)	7.2 (3.6)	7.2 (3.5)	
	n (NA)	45 (0)	35 (0)	80 (0)	
TBSA class (%)	0–5%	14 (31.11%)	13 (37.14%)	27 (33.75%)	0.82
	5–10%	20 (44.44%)	15 (42.86%)	35 (43.75%)	
	>10%	11 (24.44%)	7 (20.0%)	18 (22.50%)	
Early medication change	24 h	45 (100%)	0 (0%)	45 (56.25%)	<0.001
	48 h	0 (0%)	35 (100%)	35 (43.75%)	

^a^ *p*-values refer to the Wilcoxon test for continuous variables and to Fisher’s exact test for categorical variables (α = 0.05).

**Table 3:** Descriptive statistics for total healing time (n = 80). Group A refers to early dressing with NaOCl solution 0.05%; Group B refers to dressing with alginogel. Mean difference [95% Confidence Interval, CI] is reported along with *p*-value from a Welch *t*-test.

Variable	Parameter	Treatment		Total
		Group A n = 45 (56.25%)	Group B n = 35 (43.75%)	
Healing time (days)	Min/Max	9.0/27.0	6.0/16.0	6.0/27.0
	Med [IQR]	17.0 [15.0; 20.0]	11.0 [9.5; 13.0]	14.5 [11.0; 18.0]
	Mean (std)	17.4 (4.0)	11.2 (2.6)	14.7 (4.6)
	n (NA)	45 (0)	35 (0)	80 (0)
	Mean diff. [95% CI]	6.17 [4.71–7.63]		*p* < 0.001

**Table 4:** Local complications and dressing management outcomes in the study cohorts (n = 80). Group A refers to early dressing with NaOCl solution 0.05%; Group B refers to dressing with alginogel. Percentages are calculated by column.

Variable	Category	Treatment (n = 80)		Total	*p*-Value ^a^
		Group A n = 45 (56.25%)	Group B n = 35 (43.75%)		
NERDS	Yes	45 (100.0%)	5 (14.29%)	50 (62.5%)	<0.001
	No	0 (0.0%)	30 (85.71%)	30 (37.5%)	
Inflammation	Absent	0 (0.0%)	3 (8.57%)	3 (3.75%)	<0.001
	Moderate	2 (4.44%)	25 (71.43%)	27 (33.75%)	
	Intense	43 (95.56%)	7 (20.0%)	50 (62.5%)	
Exudate	Scarce	6 (13.33%)	5 (14.29%)	11 (13.75%)	0.004
	Moderate	17 (37.78%)	24 (68.57%)	41 (51.25%)	
	Medium	22 (48.89%)	5 (14.29%)	27 (33.75%)	
	High	0 (0.0%)	1 (2.85%)	1 (1.25%)	
Maceration	None	3 (6.67%)	33 (94.29%)	36 (45.0%)	<0.001
	<30%	30 (66.67%)	2 (5.71%)	32 (40.0%)	
	>30%	12 (26.66%)	0 (0.0%)	12 (15.0%)	
Borders	Neat	3 (6.67%)	14 (40.0%)	17 (21.25%)	<0.001
	Irregular	41 (91.11%)	21 (60.0%)	62 (77.5%)	
	Macerated	1 (2.22%)	0 (0.0%)	1 (1.25%)	
Subsequent medication	No change	1 (2.22%)	35 (100.0%)	36 (45.0%)	<0.001
	Alginogel	3 (6.67%)	0 (0.0%)	3 (3.75%)	
	Collagenase + Sodium Hyaluronate	10 (22.22%)	0 (0.0%)	10 (12.5%)	
	Sodium Hyaluronate Cream	4 (8.89%)	0 (0.0%)	4 (5.0%)	
	Gelling Fiber with Silver	27 (60.0%)	0 (0.0%)	27 (33.75%)	

^a^ *p*-values refer to Fisher’s exact tests for categorical variables (α = 0.05).

**Table 5:** VSS total score and sub-scores in the study cohorts (n = 80). Group A refers to early dressing with NaOCl solution 0.05%; Group B refers to dressing with alginogel. Percentages are calculated by column.

Variable	Category	Treatment (n = 80)		Total	*p*-Value ^a^
		Group A n = 45 (56.25%)	Group B n = 35 (43.75%)		
VSS total score	Min/Max	1.0/7.0	0/3.0	0/7.0	
	Med [IQR]	4.0 [4.0; 5.0]	1.0 [1.0; 2.0]	3.0 [1.8; 5.0]	
	Mean (std)	4.4 (1.2)	1.4 (0.9)	3.1 (1.8)	
	n (NA)	45 (0)	35 (0)	80 (0)	
	Mean diff. [95% CI]	2.95 [CI 95%: 2.33–3.23]			*p* < 0.001
Vascularization	Normal	0 (0.0%)	5 (14.29%)	5 (6.25%)	<0.001
	Intense purpura	1 (2.22%)	0 (0.0%)	1 (1.25%)	
	Pink	11 (24.44%)	27 (77.14%)	38 (47.50%)	
	Red	33 (73.35%)	3 (8.57%)	36 (45.00%)	
Pigmentation	Hyper-pigmented	38 (84.45%)	8 (22.86%)	46 (57.5%)	<0.001
	Under-pigmented	6 (13.33%)	2 (5.71%)	8 (10.0%)	
	Normal	1 (2.22%)	25 (71.43%)	26 (32.5%)	
Elasticity	Flexible	17 (37.78%)	5 (14.29%)	22 (27.50%)	<0.001
	Rather flexible	14 (31.11%)	0 (0.0%)	14 (17.50%)	
	Rather rigid	2 (4.44%)	0 (0.0%)	2 (2.50%)	
	Normal	12 (26.67%)	30 (85.71%)	42 (52.50%)	
Scar height	<2 mm	16 (35.56%)	0 (0.0%)	16 (20.0%)	<0.001
	No elevation	29 (64.44%)	35 (100.0%)	64 (80.0%)	

^a^ *p*-values refer to Wilcoxon test for quantitative variables and to Fisher’s exact tests for categorical variables (α = 0.05).

## Data Availability

The original contributions presented in this study are included in the article/Supplementary Material. Further inquiries can be directed to the corresponding author.

## Supplementary Materials

The following supporting information can be downloaded at: https://www.mdpi.com/article/10.3390/children13080994/s1, Table S1: Healing time according to burn depth and initial topical treatment. Values are presented as mean ± standard deviation (SD). Results for superficial partial-thickness (IIA) and deep partial-thickness (IIB) burns should be interpreted cautiously because of the limited number of patients, particularly in Group B. No patient with a deep-partial-thickness (IIB) burn was included in Group B; therefore, a comparison between treatment groups was not possible for this subgroup. Table S2: Early surgery and subsequent treatment according to burn depth and treatment group. Distribution of early surgical intervention within 72 h and subsequent medication strategies, stratified by burn depth and treatment group. Group A refers to NaOCl-based treatment, whereas Group B refers to enzyme alginogel treatment. Data are reported as number and percentage within each outcome category; Figure S1: Healing time according to treatment group and burn depth. Box-plot showing the distribution of clinical healing time across burn depth categories and treatment groups. Group A refers to NaOCl-based treatment, whereas Group B refers to enzyme alginogel treatment. Across all burn depth categories, patients treated with enzyme alginogel showed shorter healing times than those treated with NaOCl-based treatment.

## Abbreviations

The following abbreviations are used in this manuscript:

TBSA	Total Body Surface Area
NaOCl	Sodium Hypochlorite
SoC	Standard Of Care
ED	Emergency Department
VSS	Vancouver Scar Scale
SD	Standard Deviation
AIC	Akaike Information Criterion
CI	Confidence Interval
