# Supplementary material for: Early Enzyme-Alginogel Treatment Improves Healing and Reduces Complications, Scarring, and Healthcare Burden in Children with Mild-to-Moderate Burns: A Retrospective Real-World Study
Source: Children (Basel). 2026 Jul 27;13(8):994. doi: 10.3390/children13080994 (PMC13510530; doi:10.3390/children13080994)
Supplement: Supplementary file 1 [file children-13-00994-s001.zip › children-4429106-supplementary.pdf]

Supplementary tables

Table S1. Healing time according to burn depth and initial topical treatment. Values are presented as mean ± standard deviation (SD). Results for superficial partial-thickness (IIA) and deep partial-thickness (IIB) burns should be interpreted cautiously because of the limited number of patients, particularly in Group B. No patient with a deep partial-thickness (IIB) burn was included in Group B; therefore, a comparison between treatment groups was not possible for this subgroup.

**Burn depth**	**Group A (n)**	**Healing time, mean ± SD (days)**	**Group B (n)**	**Healing time, mean ± SD (days)**
First-degree superficial	25	16.4 ± 3.3	16	9.7 ± 2.0
Superficial partial-thickness (IIA)	6	14.5 ± 3.0	2	13.5 ± 0.7
Deep partial-thickness (IIB)	4	20.0 ± 2.6	0	—
Full-thickness (III)	10	20.5 ± 4.4	17	12.4 ± 2.3

Table S2. Early surgery and subsequent treatment according to burn depth and treatment group. Distribution of early surgical intervention within 72 h and subsequent medication strategies, stratified by burn depth and treatment group. Group A refers to NaOCl-based treatment, whereas Group B refers to enzyme alginogel treatment. Data are reported as number and percentage within each outcome category.

**Burn depth**		**Grade 1**		**Grade 2**		**Grade 3**	
**Treatment group**		*A*	*B*	*A*	*A*	*B*	*A*
Early surgery (72h)	Yes	9 (42.86%)	0 (0%)	3 (14.29%)	0 (0%)	9 (42.86%)	0 (0%)
	No	16 (27.12%)	16 (27.12%)	7 (11.86%)	2 (3.39%)	1 (1.69%)	17 (28.81%)
Subsequent medication	No change	0 (0%)	16 (44.44%)	1 (2.78%)	2 (5.56%)	0 (0%)	17 (47.22%)
	Alginogel	1 (33.33%)	0 (0%)	1 (33.33%)	0 (0%)	1 (33.33%)	0 (0%)
	Collagenase + Sodium Hyaluronate	6 (60.00%)	0 (0%)	3 (30.00%)	0 (0%)	1 (10.00%)	0 (0%)
	Sodium Hyaluronate Cream	4 (100.00%)	0 (0%)	0 (0%)	0 (0%)	0 (0%)	0 (0%)
	Gelling Fiber with Silver	14 (51.85%)	0 (0%)	5 (18.52%)	0 (0%)	8 (29.63%)	0 (0%)

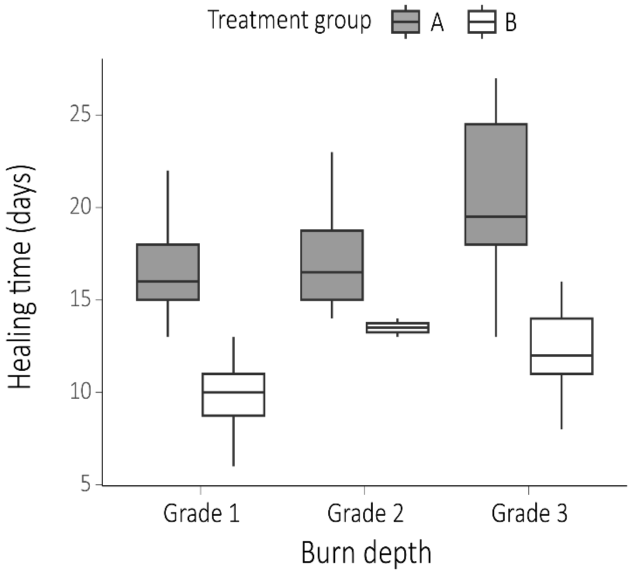

Figure S1. Healing time according to treatment group and burn depth. Box-plot showing the distribution of clinical healing time across burn depth categories and treatment groups. Group A refers to NaOCl-based treatment, whereas Group B refers to enzyme alginogel treatment. Across all burn depth categories, patients treated with enzyme alginogel showed shorter healing times than those treated with NaOCl-based treatment.
